# Transcriptome analysis of creeping bentgrass exposed to drought stress and polyamine treatment

**DOI:** 10.1371/journal.pone.0175848

**Published:** 2017-04-26

**Authors:** Yingmei Ma, Vijaya Shukla, Emily B. Merewitz

**Affiliations:** Department of Plant, Soil and Microbial Sciences, Michigan State University, East Lansing, Michigan, United States of America; Clemson University, UNITED STATES

## Abstract

Creeping bentgrass is an important cool-season turfgrass species sensitive to drought. Treatment with polyamines (PAs) has been shown to improve drought tolerance; however, the mechanism is not yet fully understood. Therefore, this study aimed to evaluate transcriptome changes of creeping bentgrass in response to drought and exogenous spermidine (Spd) application using RNA sequencing (RNA-Seq). The high-quality sequences were assembled and 18,682 out of 49,190 (38%) were detected as coding sequences. A total of 22% and 19% of genes were found to be either up- or down-regulated due to drought while 20% and 34% genes were either up- or down- regulated in response to Spd application under drought conditions, respectively. Gene ontology (GO) and enrichment analysis were used to interpret the biological processes of transcripts and relative transcript abundance. Enriched or differentially expressed transcripts due to drought stress and/or Spd application were primarily associated with energy metabolism, transport, antioxidants, photosynthesis, signaling, stress defense, and cellular response to water deprivation. This research is the first to provide transcriptome data for creeping bentgrass under an abiotic stress using RNA-Seq analysis. Differentially expressed transcripts identified here could be further investigated for use as molecular markers or for functional analysis in responses to drought and Spd.

## Introduction

Creeping bentgrass (*Agrostis stolonifera*) is a perennial, cool-season turfgrass and forage species that is susceptible to various abiotic stresses, particularly drought stress. Drought stress causes a cascade of physiological changes in creeping bentgrass leading to inhibition of photosynthesis and disruption in numerous cellular components and processes [1]. As a turfgrass under high input management conditions, creeping bentgrass primarily relies on drought tolerance mechanisms, as escape or avoidance mechanisms can be restricted by those turfgrass management practices. For instance, avoidance of drought by deep rooting is often restricted by severely low mowing height and the plant’s natural regulation of the root to shoot ratio [2]. Several tolerance mechanisms are thought to be of major importance in grass species including antioxidants and late embryogenesis abundant proteins [3–6]. Despite current knowledge of various tolerance pathways, major methods to improve drought tolerance of creeping bentgrass are still needed and many biochemical pathways associated with stress tolerance are poorly investigated in turf or forage grass species.

Polyamine (PA) biosynthesis has been shown to be involved in abiotic stress tolerance. PAs are aliphatic, amine compounds that play a major role in regulating numerous biological processes. Spermidine (Spd), spermine (Spm), and putrescine (Put) are three major PAs in plants, all of which are involved in signaling for cell growth, development, and stress responses [7]. Exogenous PA application plays a protective role in reducing drought stress symptoms in various plant species. In creeping bentgrass, pretreatment with Spd improved photochemical efficiency during drought and reduced lipid peroxidation [8]. Spm application enhanced antioxidant enzyme activities in creeping bentgrass during drought [9]. Spm also alleviated drought stress of white clover (*Trifolium repens*) by increasing production of sucrose, fructose, and dehydrins [10]. PAs may be involved in biosynthesis of auxin, abscisic acid (ABA), ethylene, and their transcription factors as well as cross-talking with reactive oxygen species (ROS) in Arabidopsis via over expressed endogenous Put and Spm [11]. Our study takes a chemical priming approach to determine the effects of PA on the transcriptome changes for drought tolerance. How PAs may be associated with lipid peroxidation, antioxidant activities, carbohydrates, or other tolerance mechanisms has not yet been fully elucidated. Transcriptome analysis of creeping bentgrass treated with Spd will improve our understanding of the gene changes associated with PA pre-treatment during drought stress.

Creeping bentgrass is an allotetraploid species (2n = 4x = 28) comprised of two A_2_A_2_ and A_3_A_3_ subgenomes [12, 13]. Heterozygosity is often problematic for transcriptome studies using hybridization based techniques. The vegetative samples we used here for RNA-Seq do not rely on hybridization and did not go through meiosis. This type of tissue is more reliable for plant species with complex genomes [14]. Expressed sequence tags (ESTs) are available in the NCBI database, which have largely been generated for marker development and molecular map construction [12, 15–18]. Currently, approximately 21,545 ESTs (as of Jan 2017) are in the NCBI EST database, of which only 132 ESTs are associated with research aimed to evaluate creeping bentgrass for drought responses. A greater number of ESTs for creeping bentgrass for drought stress responses are needed in the database to serve as a resource for turf or forage grass scientists.

Gene expression changes on a whole transcriptome level associated with drought stress or drought protective compounds of turfgrass species have not been well-studied. RNA-Seq technology has been used in many other plant species and is powerful for plants that are not model species, have complex genomes, or do not have a fully sequenced genome [14]. Some examples include sweet potato (*Ipomoea batatas*) [19], watermelon (*Citrullus lanatus*) [20], and white lupin (*Lupinus albus*) [21]. So far, RNA-Seq has been used in turfgrass species for a better understanding of fungal pathogen interactions with creeping bentgrass [22], morphological attributes of Kentucky bluegrass (*Poa pratensis*) [23] and salt stress of Kentucky bluegrass [24]. To our knowledge, this is the first report of leaf transcriptome analysis by RNA-Seq technology in creeping bentgrass subjected to an abiotic stress. Additionally, little information is available for transcriptome changes in response to PA application in crop species. Therefore, this work will serve as a valuable resource for future studies to improve drought tolerance in economically important turfgrass species and for better understanding the role of PAs in drought tolerance. The objectives of the study were to perform transcriptomic analysis by RNA-Seq to detect DE genes involved in creeping bentgrass under drought stress and PA application and to better interpret their biological meanings using gene ontology and enrichment analysis.

## Materials and methods

### Plant materials and growth conditions

The plants, experimental conditions, and treatments utilized for this study are described in more detail in Shukla et al. [8]. Briefly, creeping bentgrass ‘Penn-G2’ plants were pretreated with 500 μM L^-1^ Spd. Experimental treatments included well-watered control plants without Spd treatment (WC), well-watered plants with Spd treatment (WS), drought control plants without Spd treatment (DC), and drought-stressed plants with Spd treatment (DS). One day after, Spd treatment, half of the plants were subjected to drought stress with full water withholding for 12 d, which resulted in the soil water content (SWC) reaching 5%. Well-watered plants were maintained at approximately 25 to 28% SWC. Leaf tissue from each treatment was collected on 5 d of drought and was used for RNA-Seq analysis.

### RNA isolation and cDNA synthesis

Plants were sampled for RNA-Seq analysis after 5 d of drought stress at the same level of SWC (5%), where Spd treated plants had a significantly greater photochemical efficiency. A total of 30 mg of frozen leaf tissue was homogenized in liquid nitrogen and total RNA was isolated using an RNeasy Mini Kit (Qiagen, Valencia, CA), as directed by the manufacturer’s instruction. Any contaminated genomic DNA was removed by using RNase free DNase set (Qiagen). RNA quality was determined on a bioanalyzer (2100; Agilent Technologies, Santa Clara, CA). RNA concentration was quantified using a nanodrop (Thermal Scientific, Wilmington, DE). RNA (1μg) was used for cDNA synthesis using iScript cDNA Synthesis Kit (Bio-Rad, Hercules, CA) according to the manufacturer’s instruction.

### cDNA sequencing and assembly

The cDNA samples were divided into four treatment groups with their biological replications: watered controls (WC1, WC2, WC3, and WC4), watered plants treated with Spd (WS1, WS2, WS3, and WS4), drought stress without Spd (DC2, DC3, and DC4), and drought stressed plants treated with Spd (DS1, DS2, DS3, and DS4). A total of 15 cDNA samples were used for library preparation and subjected to cDNA sequencing (Illumina HiSeq 2500, San Diego, CA). Illumina TruSeq RNA library preparation and quality control, Illumina HiSeq 2500 Rapid 2x150-bp sequencing, transcriptome quality control, assembly, alignment, scaffolding, and annotation were performed at the genomics core facility of Michigan State University (East Lansing, MI). Two lanes of sequencing were used to generate 260–300 million read pairs which provided coverage of approximately 15 times of the creeping bentgrass genome that is approximately 2800 Mbp [25]. This provided good coverage for both de novo assembly (S1 Table) and DE gene analysis.

Data quality control was performed by removing library adapter sequences, random hexamer priming bases, and low quality base calls. De novo transcriptome assembly was carried out with Trinity assembler (R20140413p) [26]. Input reads were normalized as fragments per kilobase of transcript per million mapped reads (FPKM) to reduce bias from highly abundant transcripts reads. Initial output of Trinity was a set of approximately 500,000 contigs (S2 Table). All input reads were aligned to this set of contigs to produce an abundance estimation of each using Bowtie in Trinity. Contigs with extremely low numbers of reads mapped to them suggested they were artifacts and were filtered out. The N50 value, based on the longest isoform per ‘gene’, was 1562 bp.

### Differentially expressed gene analysis

**Fig 1:**
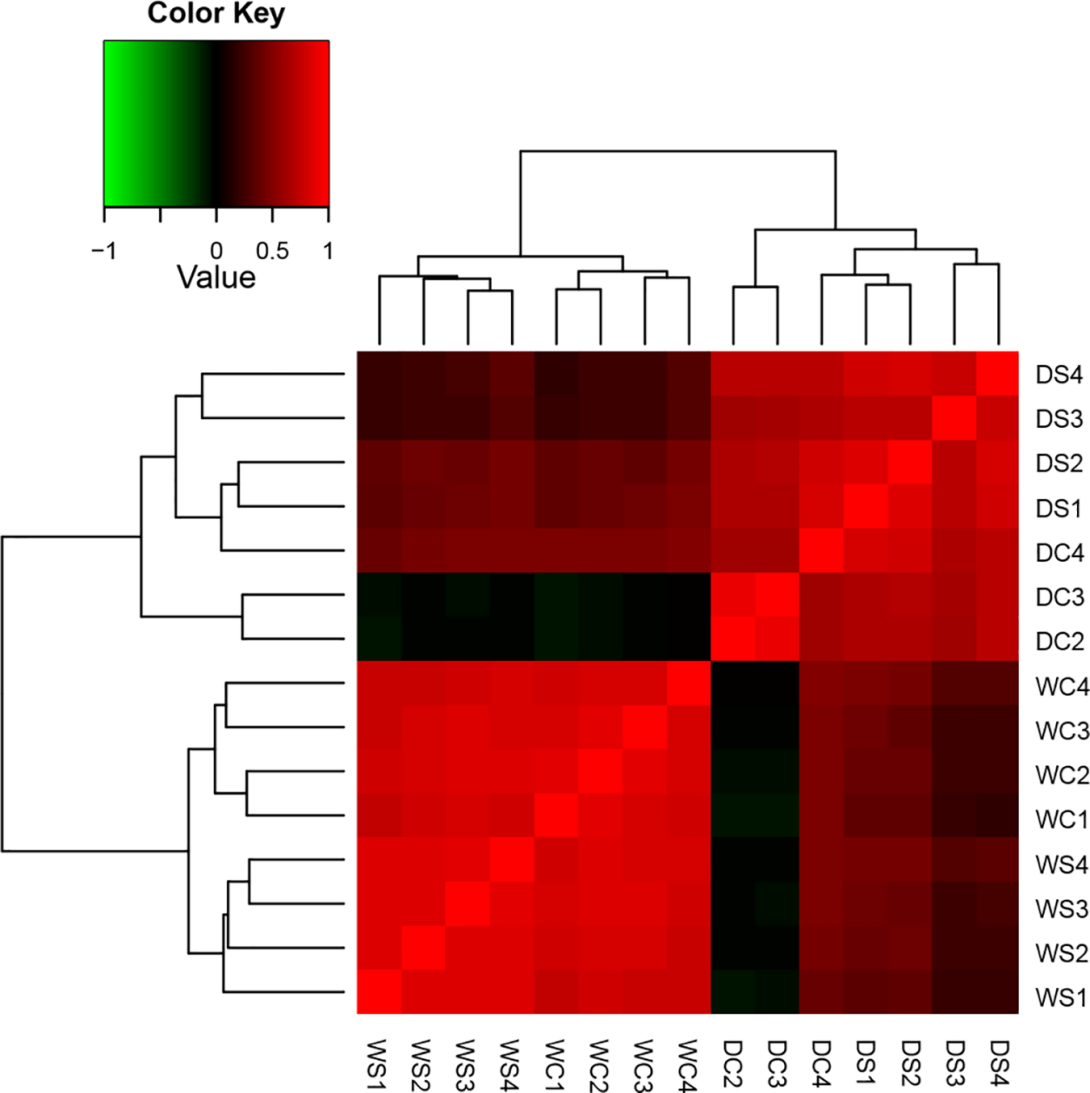
Pairwise correlation analysis of gene expression between biological replications for all samples. These gene expression in creeping bentgrass was exposed to the following experimental treatments. WS = watered plants treated with spermidine (Spd); WC = watered control plants (no Spd); DC = drought treated control plants (no Spd); DS = Drought plants treated with Spd. The dendrogram on the top was divided into two parts by representing water or drought treatment with all biological replications. The dendrogram on the left was separation of water or drought treatment with all biological replications.

**Fig 2:**
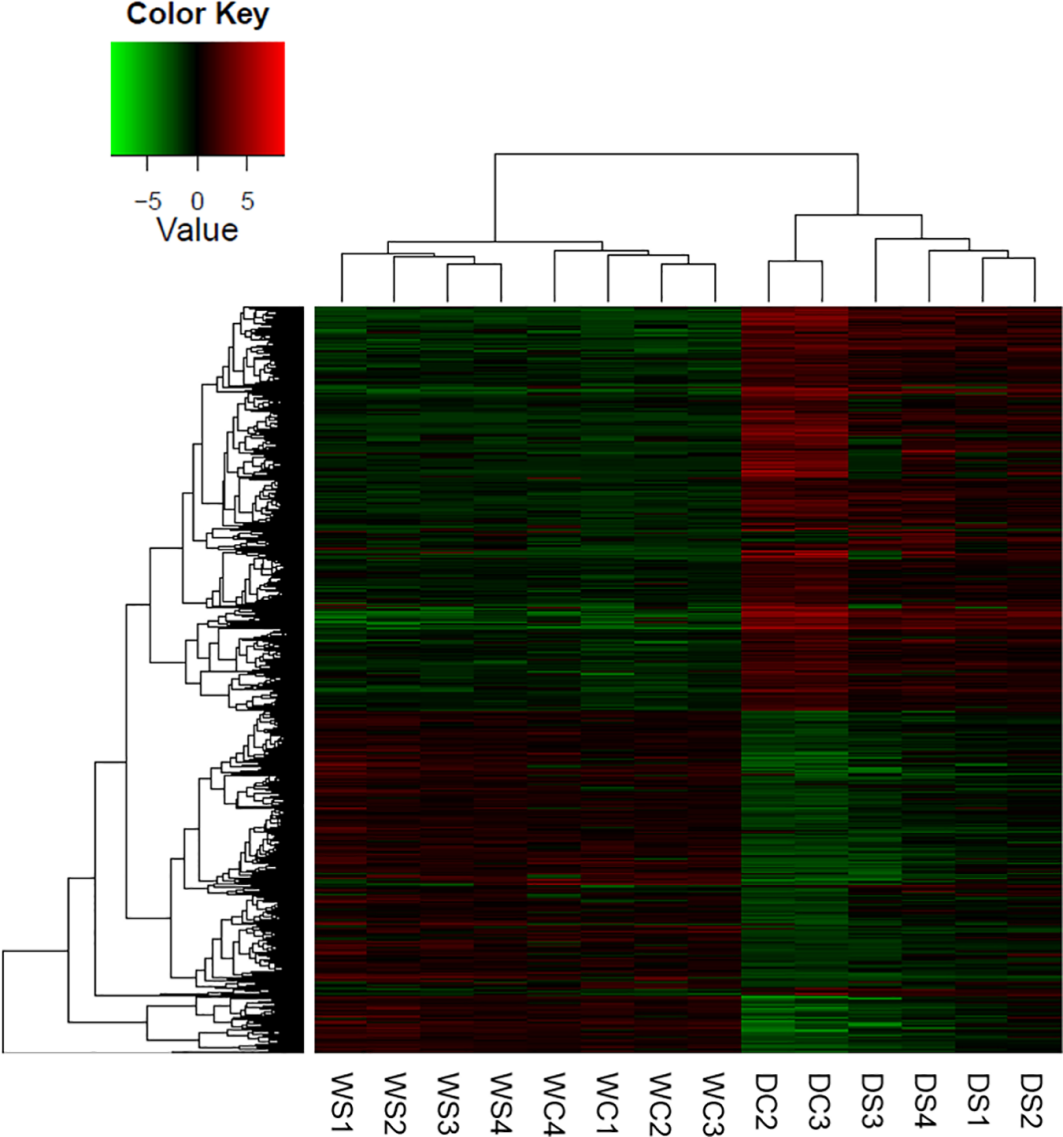
Heat map of all differentially expressed genes in creeping bentgrass. These differentially expressed genes were from 14 samples with all biological replications exposed to the following experimental treatments. WS = watered plants treated with spermidine (Spd); WC = watered control plants (no Spd); DC = drought treated control plants (no Spd); DS = Drought plants treated with Spd. The dendrogram on the top was divided into two parts by representing water or drought treatment with all biological replications. The dendrogram on the left was separation of all transcripts involved in all possible biological processes. The processes that most relevant to drought and Spd were described in discussion part.

Pairwise analysis of DE genes was conducted using the Trinity toolset in Bioconductor package [27]. Sample DC4 was excluded from further analysis because its expression pattern was more similar to the DS samples than the other two DC replicates based on initial sample correlation analysis (Figs 1 and 2). Other than this sample, the correlation analysis revealed consistency among biological replicates. All input reads were aligned to the set of filtered transcripts with Bowtie in Trinity and the abundance of each was estimated with RNA-Seq expectation-maximization (RSEM) which computes ‘gene-level’ estimates as a proxy for the gene [28–29]. In order to compare expression level of DE genes across treatments, each pair of DE genes was analyzed in turn (WC vs WS, DC vs DS, WC vs DC, WS vs DC, and WS vs DS) using EdgeR (version 2.14) [30]. A web-based tool called Vennt (version 0.8.1) [31] was utilized for examining lists of DE genes that are either up- or down-regulated defined at a false discovery rate threshold (FDR) of 0.001 and log_2_ fold change larger than 2.0.

### Annotation analysis

Trinity filtered contigs (80,996; S3 Table) were subjected to a process that detects coding sequences (CDS) using Transdecoder in Trinity and those CDS were blasted using BLASTX against the SwissProt reference protein database [32] to identify known proteins for functional annotation. Gene ontology (GO) and kyoto encyclopedia of genes and genomes (KEGG) pathway functional enrichment analyses were performed using Fisher’s exact test module in Blast2GO software (version 2.7.2) [33–34]. The blast expectation value (E-value) was 0.001 and the highest scoring paring value was 33. GO is widely used to categorize over-represented genes for biological processes using annotation standards based on the top BLAST hits and identified domains [35–36]. Enriched transcripts represented by number of tested sequences over reference sequences were provided where they were identified. DE genes were constructed in a heatmap using R package (Rx64, 3.3.2.3). Biological process (BP), molecular function, and cellular components were produced from GO where one or more levels of assemblies in molecular functions are used to describe a BP [37]. KEGG pathway provides a comprehensive way of interpreting the network of high throughput sequence data that is complementary to the currently published molecular biology where enzymes that encoded by genes in the genome are reconstructed into a diagram by direct mapping of the GO with their enzyme codes for particular biochemical pathway [38–41]. All genes detected in the KEGG pathways exposed to drought and Spd were summarized in a diagram.

### qRT-PCR confirmation

cDNA was synthesized from the same RNA samples that were used in RNA-Seq analysis for qRT-PCR analysis. Fifty-four primer pairs were designed from RNA-Seq sequences with TaqMan MGB Quantification methods (Primer Express 3.0; S4 Table). Twenty out of 54 primer pairs were screened, selected, and subjected to qRT-PCR. PCR reactions were conducted using a 2X Power SYBR Green PCR Master Mix (Life Technologies Inc, Carlsbad, CA) in a fast real-time PCR system (7900 HT; Applied Biosystems, Foster City, CA). Actin was used as a reference gene for data normalization.

### Statistical analysis and data availability

Statistical analysis of RNA-Seq results was described above. For qRT-PCR, data analysis was performed using a comparative cycles of threshold (CT) method to calculate the fold changes of each gene. The correlation between RNA-Seq results and qRT-PCR expression was analyzed using PROC COR procedure in SAS (SAS 9.4 for Windows, Cary, NC) to get Pearson’s correlation coefficients. The raw cDNA reads were deposited in sequence read archives (SRA). The transcriptome shotgun assembly project has been deposited at GenBank under the accession GEUC00000000. The version described in this paper is the first version, GEUC01000000. In order to meet the guidelines set by the transcriptome shotgun assembly from NCBI, two contigs required trimming due to adapter contamination. The first 25 bases of the 5’ end of c211554_g1_i7 were trimmed off. The last 38 bases of the 3’ end of c206014_g1_i3 were trimmed off. Gene expression analysis was not updated to reflect this change as the modification to these contigs had a marginal effect on the FPKM values computed and, to an even lesser extent, the differential gene expression.

## Results

### Sequence assembly

All reads were aligned to the sets of filtered transcripts (80,996) and alignment efficiency was calculated as a percentage for number of aligned reads over the filtered read pairs in assembly (S5 Table). All alignment efficiencies were about 60% while DS2 had 74% of its input reads aligned. In model organisms such as Arabidopsis, researchers may obtain higher percentage of alignments mapped, but for a de novo assembly without reference genome for which we have created a reference transcriptome; these results are to be expected. Open reading frames (ORF) and translated peptide sequences were identified in Trinity in which showed 80,996 transcripts as coming from 51,571 genes. Of 80,996 transcripts, 49,190 ORFs meeting minimal criteria were identified. 18,682 of these were complete coding sequences (Accessions deposited in GenBank).

### Differential expression and gene ontology

**Fig 3:**
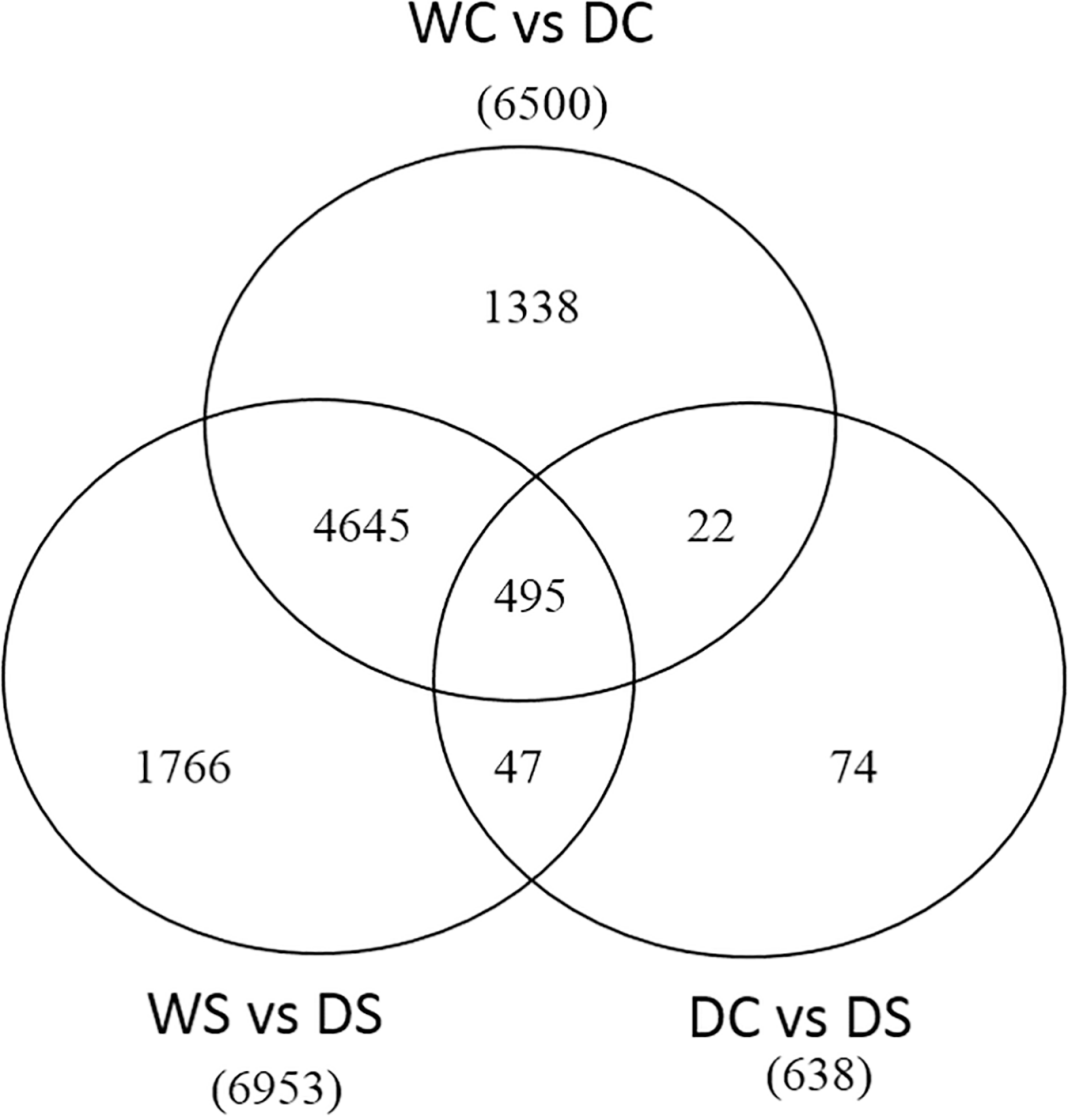
Venn diagram for all differentially expressed (DE) genes in creeping bentgrass. These differentially expressed (DE) genes were from exposure to the following experimental treatments. WS = watered plants treated with spermidine (Spd); WC = watered control plants (no Spd); DC = drought treated control plants (no Spd); DS = Drought plants treated with Spd. DE genes were quantified at false discovery rate threshold (FDR) of 0.001 and log_2_ fold change larger than 2. Total DE genes for each comparison are shown in parenthesis.

A total of 9,109 transcripts were identified as DE genes in one or more of the pairwise comparisons (Fig 3). Under well-watered conditions, the addition of spermidine (Spd) had little effect on transcription. This was also made clear from the heatmap of gene expression for all DE genes where the distance between these two groups (WC vs WS) was not significantly different from the distance between replicates within each group (Fig 2). The heatmap also indicates the overall effect of drought stress on transcription and allows for visualization of how Spd moderated the effects of drought stress on the transcriptome. A large change of the transcriptome occurred in creeping bentgrass in response to drought stress. A total of 6,504 genes were either up- or down-regulated when comparing well-watered to drought stressed plants (Fig 3). Gene ontology (GO) and enrichment analysis identified 741 biological processes, 197 cellular components, and 334 molecular functions (S6 Table). Only the GOs most relevant to drought stress and Spd application are focused on in the discussion.

### Differentially expressed genes due to drought stress

**Fig 4:**
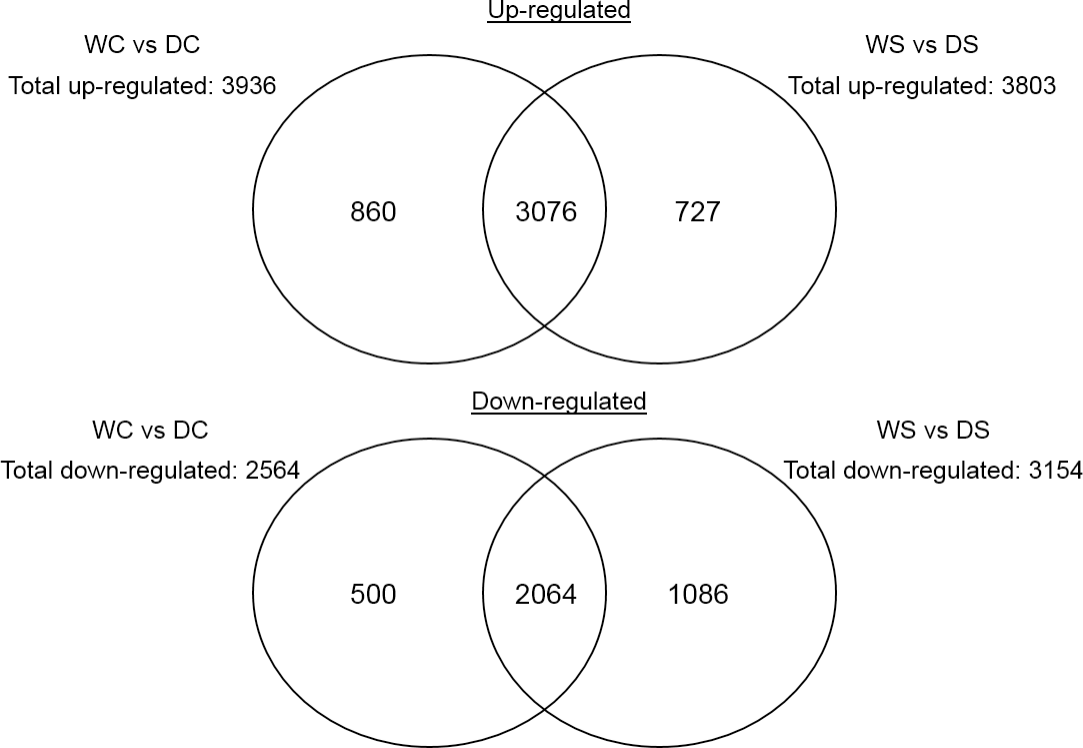
Venn diagram for genes that up- and down-regulated in creeping bentgrass. These differentially expressed (DE) genes were exposed to the following experimental treatments. WS = watered plants treated with spermidine (Spd); WC = watered control plants (no Spd); DC = drought treated control plants (no Spd); DS = Drought plants treated with Spd. DE genes were quantified at false discovery rate threshold (FDR) of 0.001 and log_2_ fold change larger than 2. Total DE genes for each comparison are shown in parenthesis.

**Fig 5:**
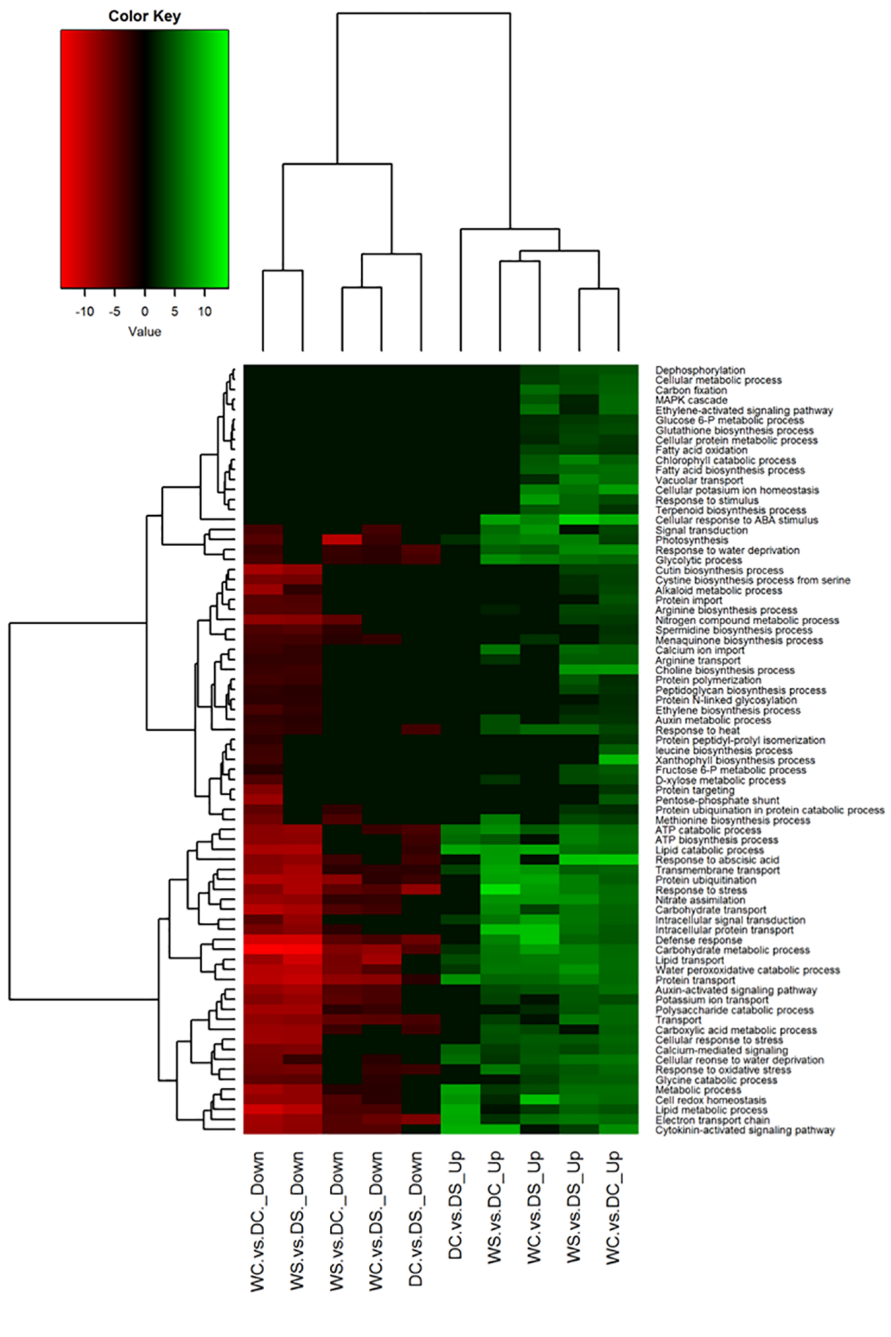
Heat map with clusters for differentially expressed (DE) genes at log_2_ fold scale in creeping bentgrass. These DE genes were exposed to the following experimental treatments. WS = watered plants treated with spermidine (Spd); WC = watered control plants (no Spd); DC = drought treated control plants (no Spd); DS = Drought plants treated with Spd. The dendrogram on the top was divided into two parts by representing up- or down- regulation of the genes under each type of treatment comparisons. The dendrogram on the left was separation of different biological processes under different type of treatment comparisons. The bottom scale is pairs of different treatment comparisons.

When compared between drought and well-watered samples (WC vs DC), 22% (860 out of 3936) transcripts were up-regulated while 500 genes out of 2564 (20%) were down-regulated (Fig 4). In response to drought, up-regulated DE genes were identified to encode heat stress transcription factor (5.9 fold) in stress response (GO: 0006950; FDR<0.0001), probable peroxygenase 4 (10.1 fold) associated with response to abscisic acid (ABA; GO: 0009737; FDR<0.0001), and tryptophan synthase beta chain 2 (5.7 fold) in oxidative stress (GO: 0006979; FDR<0.0001; Fig 5). In addition, genes encoding aminocyclopropane-1-carboxylate oxidase 2 (2.5 fold) associated with ethylene biosynthesis (GO:0009693; FDR = 0.0074) were identified to be up-regulated for hormone related leaf senescence due to drought stress (Fig 5).

Enriched transcripts associated with amino acid biosynthesis were identified due to drought. Up-regulation of a DE gene encoding a probable electron transfer flavoprotein-quinone oxidoreductase (3.2 fold) in nitrogen compound metabolic process (GO:0006807; FDR<0.0001) was detected. Additionally, up-regulation occurred for DE genes encoding homocysteine methyltransferase (3.4 fold) in methionine biosynthesis (GO:0009086; FDR = 0.0105), acetylornithine deacetylase (3.8 fold) in arginine biosynthesis (GO:0006526; FDR = 0.0261), probable serine acetyltransferase (3.6 fold) in cysteine biosynthesis from serine (GO:0006535; FDR<0.0001), and 3-isopropylmalate dehydratase large subunit (5.1 fold) in leucine biosynthesis (GO:0009098; FDR = 0.0466). A DE gene was also detected in Spd biosynthesis (GO:0008295; FDR = 0.0007; Fig 5, S1 and S2 Figs).

Enriched transcripts associated with sugar metabolism were detected. When compared to well-watered plants, drought caused an up-regulation of beta-amylase transcripts (7.6 fold) involved in water deprivation (GO: 0009414; FDR<0.0001). Up-regulation was also identified for glucose-6-phosphate 1-dehydrogenase transcripts (4.9 fold) involved in the pentose-phosphate shunt (GO:0006098; FDR = 0.0442; Fig 5; S3 Fig).

### Differentially expressed genes due to Spd application under watered conditions

When samples without Spd treatment were compared to samples treated with Spd under well-watered conditions (WC vs WS), only 37 out of 9109 transcripts were differentially expressed. This indicates that Spd did not play a major role in regulating transcription under watered conditions in creeping bentgrass (data not shown).

### Differentially expressed genes due to drought of Spd treated plants

Comparing plants treated with Spd under well-watered to drought stressed conditions (WS vs DS), 19% (727 out of 3803) of the transcripts were up-regulated and 34% (1086 out of 3154) were down-regulated (Fig 4). These regulated transcripts were enriched in response to drought stress and were involved in many biochemical processes. For instance, Spd application caused up-regulation of genes associated with photosynthesis under drought, such as a gene encoding photosystem II 10 kDa polypeptide (3.1 fold) involved in photosynthesis (GO:0015979; FDR<0.0001; Fig 5), ribulose bisphosphate carboxylase small chain clone 512 (GO:0019253; FDR<0.0001; -9.9 fold), and phosphoenolpyruvate carboxylase (PEPC; 4.2 fold) in carbon fixation (GO:0015977; FDR<0.0001) (Fig 5 and S4 Fig).

Sugar metabolism related transcripts enrichment was affiliated with Spd application under drought stress. When comparing drought and well-watered plants, up-regulation was detected for DE genes encoding beta-amylase 1 (7.1 fold) in response to water deprivation (GO:0009414; FDR<0.0001), sucrose synthase (SS) 4 (7.5 fold) and SS4 (3 fold) involved in starch (GO:0005982; FDR<0.0001) and sucrose (GO:0005985; FDR = 0.00017) metabolism, respectively. Expression of a gene encoding galactinol-sucrose galactosyltransferase 1 (-4.5 fold) in carbohydrate metabolism (GO:0005975; FDR<0.0001) was down-regulated (Fig 5 and S5 Fig).

Transporter associated genes were detected in drought and Spd treated plants. For instance, down-regulation occurred for a gene encoding a high affinity nitrate transporter (-4.5 fold) involved in nitrate assimilation (GO:0042128; FDR = 0.004), and a calcium-binding mitochondrial carrier protein, SCaMC-1 (-5.1 fold), involved in transmembrane transport (GO:0055085; FDR<0.0001). Up-regulation for a gene encoding a bidirectional sugar transporter SWEET15 (2.9 fold), within the GO category of cellular response to stimulus (GO:0071215; FDR<0.0001), was also found (Fig 5).

Enrichment for transcripts associated with signaling was also detected. When comparing drought and well-watered plants, genes encoding a gibberellin (GA) regulated protein 2 (2.1 fold) involved in GA mediated signaling pathway (GO:0009740; FDR<0.0001) and CHD3-type chromatin-remodeling factor PICKLE (3.4 fold) involved in cytokinin-activated signaling pathway (GO:0009736; FDR = 0.018) were up-regulated (Fig 5). Expression of a gene encoding an ethylene-responsive transcription factor, ERF054, (-4.5 fold) that is involved in the ethylene-activated signaling pathway (GO:0009873; FDR<0.0001) was down-regulated (Fig 5).

DE genes related to the antioxidant system were detected. For instance, a gene encoding a cationic peroxidase SPC4 (2.2 fold) involved in hydrogen peroxide catabolic process (GO:0042744; FDR<0.0001) was up-regulated due to Spd treatment. Expression of a gene encoding a glutathione S-transferase GSTU1 (-2.8 fold), involved in cellular response to water deprivation processes (GO:0042631; FDR = 0.0125), was down-regulated due to Spd treatment (Fig 5).

### qRT-PCR validation of RNA-Seq results

**Fig 6:**
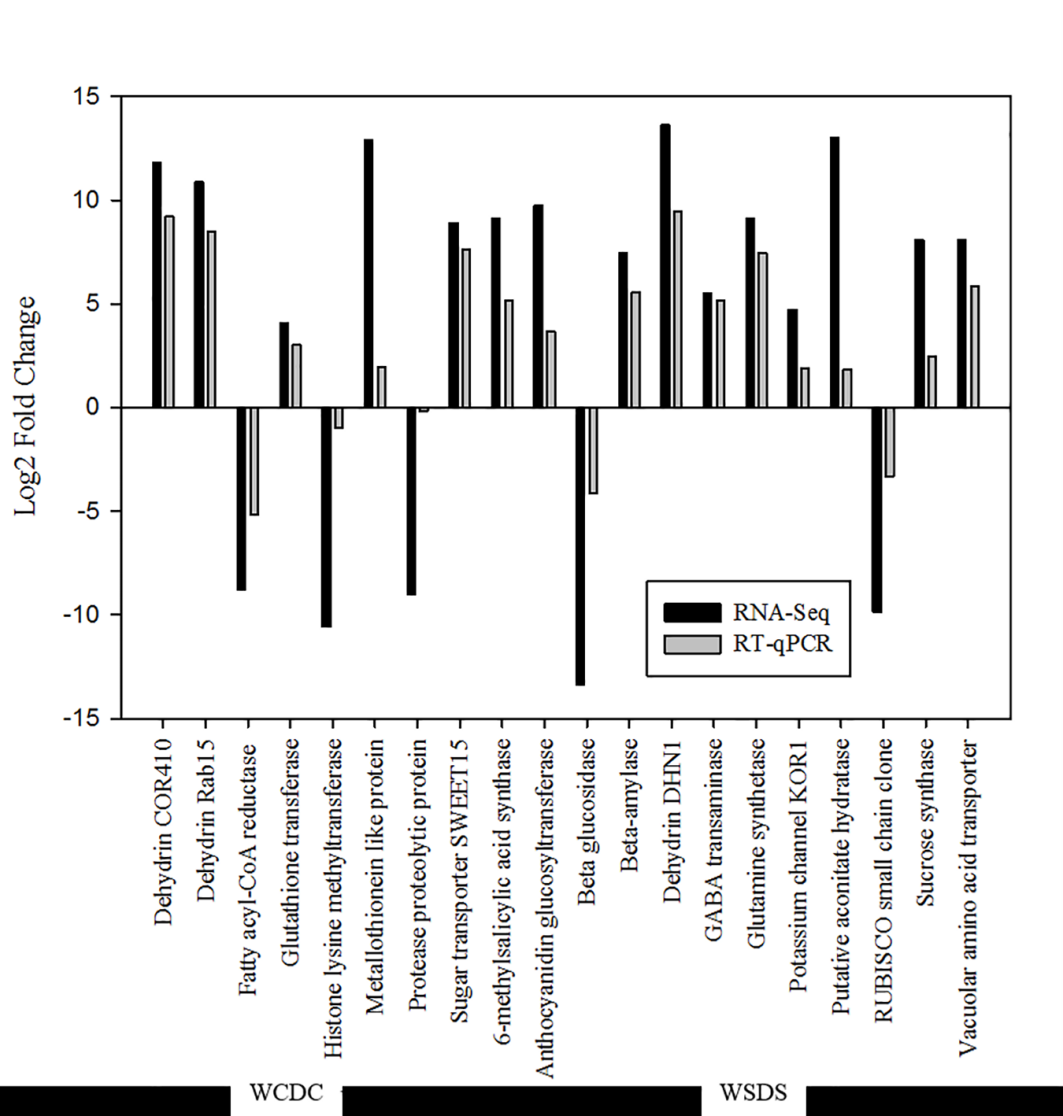
Log_2_ fold changes of genes based on RNA-Seq and qRT-PCR methods. These genes were regulated by drought stress and/or spermidine (Spd) treatment. Fold changes were calculated based on the following comparisons: WCDC, watered controls compared to drought controls, WSDS: watered plants treated with Spd compared to drought + Spd treatment.

A total of 20 genes used for qRT-PCR had 90% ± 10% of amplification efficiencies with a single dissociation peak and linearity between target cDNA and Ct values. These genes used for qRT-PCR were all consistent with the RNA-Seq results (Pearson’s *r* = 0.83, P < 0.001; Fig 6).

## Discussion

Due to the widespread availability of transcriptome data associated with drought stress in model and crop species such as in Arabidopsis and maize [42], the following includes a brief discussion of gene changes solely due to drought stress (WC vs DC) but is primarily focused on PA effects on drought tolerance. A discussion of other relevant and interesting gene changes that may be specific for creeping bentgrass is also provided. Major changes in transcriptome due to Spd treatment under watered conditions (WC vs WS) were not detected; however, to avoid negating any differences due to Spd treatment under watered conditions, this discussion will primarily focus on comparing WS to DS instead of WC to DS, in order to not confound DEG results revealed and for a more concise discussion of the results. It is also worthy to note that often transcriptome changes are not always correlated to changes in protein expression such as the discrepancies found between microarray and protein profiling of salt stress in Arabidopsis [43]. Further experimental evidence is needed to confirm the fate of the genes identified here; however, this work provides a good reference for genes of interest associated with PAs and drought stress.

### Genes differentially expressed due to drought stress

Drought-induced physiological changes are a result of numerous gene expression changes that act to alter biochemical processes to escape, avoid, or tolerate drought stress such as photosynthesis, respiration, sugar metabolism, defense pathways, and hormone signaling [44–45]. ABA and ethylene are the most closely associated hormones with drought stress perception and signaling [46]. In this study, enriched transcripts associated with ethylene were found. Drought stress caused an up-regulation of genes encoding amino-cyclopropane-carboxylate oxidase (ACC oxidase; 2.4 fold) which converts ACC into ethylene [47] and two ethylene transcription factors, APETALA2/ethylene response (RAP2-4; 3.3 fold) and ethylene response factor (ERF054; 5.6 fold). This is consistent with transcript enrichment of ethylene biosynthesis in response to drought in soybean (*Glycine max*) [48]. In Arabidopsis, RAP2-4 mediates ethylene signaling pathways by constitutively binding to ethylene and dehydration responsive elements during drought [49]. Similar to these findings, over expression of transcription factor (*ERF*) in wheat (*Triticum aestivum*) [50] and Arabidopsis [51] showed significantly higher drought and salt tolerance than wild type via accumulation of proline, maintaining redox homeostasis, reduced transpiration water loss, and lower stomatal conductance. Relatively little information regarding ethylene biosynthesis and drought stress is available for creeping bentgrass or other important turf or forage species; further investigation into ethylene responses during drought stress and how ethylene relates to PAs may be warranted for improvement of creeping bentgrass performance under drought stress.

Drought induced an up-regulation (3.5 fold) of a gene encoding ABA biosynthesis in creeping bentgrass. Up-regulation of ABA biosynthesis gene expression is expected as it can trigger stomatal closure [52–53]. ABA can also regulate molecular chaperones [54], which are a family of proteins that facilitate protein folding, reducing misfolding, stabilizing or maintaining the integrity of the cell membrane or enzymes, or preventing aggregation or disaggregation of proteins for normal function [55]. In creeping bentgrass, the expression of a gene encoding dehydrin RAB15 was up-regulated in response to drought stress. RAB15 is an ABA responsive dehydrin, which has chaperone-like functions to maintain the integrity of cell walls in wheat [56–57] and reduce water loss in a drought tolerant Bermuda grass (*Cynodon dactylon*) [4]. Another gene encoding chaperone type protein, dehydrin COR410, was up-regulated (5.1 fold) by drought in creeping bentgrass. COR410 was initially identified in the plants under cold stress while it was also induced by ABA and drought [58]. The drought induced expression of gene encoding COR410 is observed in wheat [59] and over expression of *COR410* protected cell membranes during cold stress in strawberry (*Fragaria × ananassa*) [60]. Thus, up-regulation of these ABA and drought induced molecular chaperones may be of critical importance for creeping bentgrass survival under drought stress.

Osmoprotectant production plays an important role in response to drought. One of the amino acid, proline, acts as osmotic compatible solute [61] and free radicals scavenger [62]. A gene encoding pyrroline-5-carboxylate reductase (P5CR) was up-regulated by 3.5-fold due to drought stress. P5CR is the rate limiting enzyme that catalyzes the conversion of δ^1^-pyrroline-5-carboxylate to L-proline [63]. In Arabidopsis, transcript induction of *P5CR* gene is associated with increased accumulation of proline after salt stress [64]. Site-directed mutation of *P5CR* gene feedback inhibitor showed more proline accumulation than the wild type in response to osmotic stress, which was associated with reduced malondialdehyde accumulation and osmotic stress tolerance [65]. Similarly, a potassium channel gene KOR1 was up-regulated by 4.4-fold. Arabidopsis mutant for gene encoding inward-rectifying K^+^ channel that uptakes K^+^ showed less K^+^ uptake and poor growth [66]. Thus, up-regulated gene expression on biosynthesis of osmotic protectant and K^+^ transporters may be associated with osmotic adjustment to contribute drought tolerance in creeping bentgrass.

**Fig 7:**
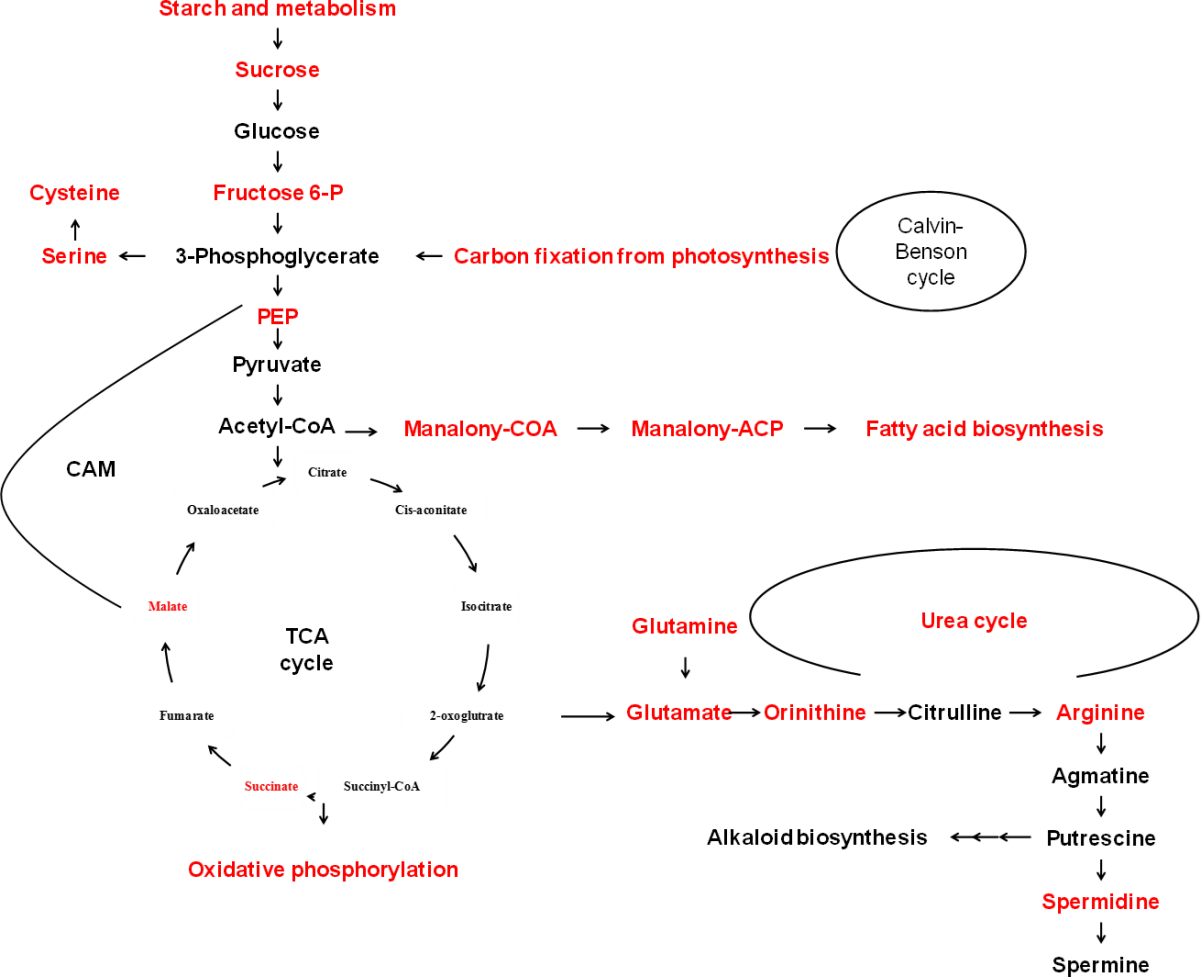
Proposed representation of TCA cycle intermediates, primary metabolites, and associated pathways. Red colored intermediates and pathways identified are for the transcripts that detected in kyoto encyclopedia of genes and genomes (KEGG) pathway to be enriched in response to Spd under drought in creeping bentgrass.

Drought stress significantly induced transcript enrichment associated with amino acid biosynthesis (WC vs DC) (Fig 7). For instance, GO categories for biosynthesis of methionine, cysteine, and leucine were enriched. More specifically, gene expression for 5-methyltetrahydropteroyltriglutamate-homocysteine methyltransferase which functions for methionine (Met) formation [67] was up-regulated. In addition to be proteinogenic of these amino acids, they are also known to be involved in biosynthesis of other compounds or being associated with stress tolerance. Met serves as a fundamental precursor for S-Adenosyl-methionine (SAM) biosynthesis and controls ethylene and PAs biosynthesis [68]. Although both ethylene and PAs are involved in drought stress tolerance in several crop species [69], the direct abiotic stress tolerance effect of methionine has not been documented. The increased transcripts in Met biosynthesis could be for increased need of Met for protein synthesis. Biosynthesis of cysteine from serine is associated with nitrogen metabolism and stress defense [70–71]. In soybean, H_2_O_2_ regulates accumulation and phosphorylation of acetyltransferase to catalyze biosynthesis of cysteine from serine. Increased cysteine accumulation due to increased activity of acetyltransferase was positively correlated with induction of glutathione, which might indicate that cysteine plays a role under oxidative stress [72]. The drought-induced enrichment of transcripts related to amino acid biosynthesis may be associated with stress defense or building stress defense proteins; however, further work on these amino acids would be required to draw such conclusions.

PA biosynthesis was also found to be affected by drought stress. Expression of genes encoding arginine decarboxylase (ADC) 1, one of the enzymes that catalyze Put formation [73], and arginine biosynthesis were up-regulated by 2.7-fold. Additionally, a gene encoding S-adenosylmethionine decarboxylase which catalyzes Spm formation was up-regulated by 6.3-fold due to drought stress (Fig 7). Put and Spm improved drought tolerance in wheat via mediating ion channels and cross talk with other phytohormones [69, 74–75]. PAs also alleviated some drought stress symptoms in Bermuda grass [76] and creeping bentgrass [8, 77]. Although Spd content accumulation in creeping bentgrass may not be a major, naturally occurring salt tolerance mechanism [77], three genes associated with Put and Spm biosynthesis were affected by drought stress. Therefore, exploitation of this stress tolerance pathway, via exogenous application of Spd, may be a viable method to promote PA-induced stress tolerance in creeping bentgrass.

### Spd effects on drought tolerance

#### Photosynthesis

Drought stress often causes photosynthesis rates to decrease due to stomatal closure, reduced sugar demands due to growth cessation, and metabolic damage of photosynthetic apparati [78]. The ability of plants to maintain photochemical health and efficiency under stressed conditions is a major drought tolerance mechanism [79]. Exogenously applied Spd in creeping bentgrass helped maintain photochemical health during drought stress compared to control plants [8]. Photochemical efficiency of the light reactions of photosynthesis is often measured by the health of photosystem II (PSII) and PSII can be damaged by drought stress [80]. Spd application significantly up-regulated (3.1 fold) expression of gene encoding a 10 kDa PSII polypeptide under drought conditions (Fig 7). Direct effect of PAs on PSII proteins revealed that low concentration (<1 mM) of Spm could bind to PSII membranes to maintain its integrity and improve photosynthetic function under stress [81]. Treatment with 50 µM Spd has been shown to increase Fv/Fm, which indicates the enhancement of PSII function [82]. Up-regulation of PSII polypeptides, which could replace damaged subunits, and possible direct protection of PSII complexes by PA treatment may benefit plants under drought stress.

Exogenously applied Spd under drought stress also appears to regulate transcripts associated with the Calvin-Benson Cycle (CBC) of photosynthesis. Expression of seven genes encoding ribulose bisphosphate carboxylase (Rubisco) small chain clone 512 (Rubisco-small) were down-regulated (-9.9 fold) in plants treated with Spd under drought condition while no regulation was found for these genes due to drought. Rubisco is comprised of large and small subunits and is a rate limiting enzyme in photosynthesis. Rubisco small subunits stabilize the large subunit to influence holoenzyme activity and substrate affinities [83–84]. Genetically engineered cyanobacteria containing genes encoding a Rubisco small subunit increased the CO_2_/O_2_ catalytic efficiency and specificity compared to non-transgenic cyanobacteria [85–86] and miRNA based gene silencing of Rubisco small subunits decreased Rubisco activity in drought resistant *Physcomitrella patens* [87]. Photorespiration activity is primarily determined by RuBP regeneration rate, CO_2_/O_2_ ratio in the chloroplast, as well as the amount and kinetics of the Rubisco holoenzyme [88–89]. Spd induced down-regulation of Rubisco small subunits could decrease Rubisco activity and could reduce both photosynthetic and photorespiratory processes in creeping bentgrass. This could reduce energy expenditure associated with these processes during drought stress. Further research that may directly elucidate the effects of PAs on these processes is needed.

Expression of a gene encoding phosphoenolpyruvate carboxylase (PEPC) was up-regulated (4.2 fold) in response to Spd treatment under drought. PEPC is well-known for the carboxylation reaction in C4 and crassulacean acid metabolism (CAM) photosynthesis [90–91]. Over-expression of the maize *PEPC* gene in rice exhibited a higher RWC and higher chlorophyll content than the wild type plants, which could indicate greater drought tolerance [92–93]. In C3 creeping bentgrass, malate formed through PEPC might be involved in providing metabolic intermediates from the tricarboxylic acid cycle for stress survival [94] and CO_2_ fixation that formed via PEPC could contribute to Calvin-Benson cycle due to stomatal closure. Whether this directly correlates to photosynthesis rates under drought may deserve further investigation.

#### Sugar metabolism

Spd treatment may also influence other sugar metabolism in creeping bentgrass exposed to drought stress based on transcript enrichment (345/160). Expression of a gene encoding beta-amylase which functions to degrade starch to form glucose and maltose [95] was up-regulated based on the comparison of WS vs DS (7.1 fold). Due to up-regulation of this starch degradation process, Spd application may increase the starch to glucose conversion process and have an effect to allocate sugar energy during drought stress.

Up-regulation was detected for genes encoding sucrose synthase (SS) 1 (3 fold) and SS4 (7.5 fold) due to drought under Spd treatment (WS vs DS). SS catalyzes sucrose formation and degradation using glucose and fructose as substrates [96]. Conversely, protein expression of SS was decreased in Spm treated white clover under drought [10]. Increased *SS* expression and SS content was also identified in transgenic plants with increased cytokinin content, which were more drought tolerant than non-transgenic plants [97–98]. Up-regulation of *SS* under drought might be activated for supplying metabolic intermediates, for respiration, or for regulating osmotic potential under drought stress; however, a better understanding of PA regulation of SS may be needed.

Similar to the regulation of *SS*, enriched transcripts (1979/1278) were identified in sucrose metabolism in response to Spd treatment. Except for the up-regulation of a gene encoding galactinol-sucrose galactosyltransferase (GSG; 5.7 fold) due to drought (WC vs DC), expression for this process was down-regulated in Spd treated plants under comparison of WS vs DS (-4.5 fold). GSG catalyzes the biosynthesis of galactinol, raffinose, and other oligosaccharides [99]. Down-regulation of *GSG* in Spd treated plants might indicate that PA treatment is altering sugar metabolism in plants. Little information is available regarding the effects of PAs on sugar relations in the plant; further research on this may be desirable.

#### Transporters

In addition to sugar metabolism, transcript enrichment (185/110) was found to be associated with carbohydrate transport in plant cells due to Spd treatment. Carbohydrate transporters play a role in photosynthate allocation; however, not much information is available about relationship between PA and sugar transporters. We found that expression of a gene encoding a bidirectional SWEET transporter was up-regulated in comparisons of WC vs DC (9.3 fold) and in WS vs DS (2.9 -fold). SWEET transporters were phloem sugar loading transporters [100], which regulate sugar movement and allocation in response to abiotic stress, plant growth, and development [101]. If Spd treatment can enhance photosynthetic health, transport of those sugars could be differential compared to plants not treated with Spd. A gene encoding plastidic glucose transporter 3 was up-regulated in Spd treated plants by 6.3-fold. Starch formed during photosynthesis can break down to form glucose to go towards growth, cellular maintenance, or stress survival. Glucose transporters play an important role in the translocation of glucose from a chloroplast and within a cell [102]. Bourque et al. [103] showed glucose transporter in tobacco is involved in programmed cell death that induced by biotic stress to minimize the stress damage. During drought stress, a more effective synthesis and movement of sugars within plant cells could be a major drought tolerance mechanism due to limited carbon acquisition due to stomatal closure and depletion of carbohydrates via respiratory processes. Further research into the effects of PAs and the effects of drought tolerance on sugar transporters is needed.

Membrane transport processes can become active for transporting organic or inorganic ions or amino acids to cope with adverse growing environment such as for osmotic adjustment [101]. Expression patterns for amino acid transporters in plants can vary under different growing environments. Enriched transcript (10/1) was found in response to Spd application under drought. Expression of DE genes encoding an amino acid permease (AAP) 3 were up-regulated by 4.2-fold in the comparisons of WS vs DS. AAP is a family of amino acid transporters that preferentially transport glutamine, asparagine, glutamate, and neutral amino acids into plant cells [104]. Glutamate acts as a precursor of proline biosynthesis to serve as an osmolyte [105]. Another study also demonstrated that accumulation of glutamine and asparagine in younger leaves of resurrection plant (*Sporobolus stapfianus*) was more desiccation tolerant than the older leaves [106]. It is possible that up-regulation of these amino acid transporters might be involved in amino acid-based osmotic regulation under drought in response to Spd treatment. Furthermore, we detected enriched transcript (21/8) for other amino acid transporters. Expression of gene encoding lysine histidine transporter (LHT) was down regulated (-8.7 fold) in both drought stressed (WC vs DC) and drought Spd treated plants (WS vs DS). LHT is an amino acid permease homolog which serves as an amino acid selective transporter, especially lysine and histidine. Transcripts encoding LHT is greatly up-regulated by ABA, amino acid, JA, and SA in ginseng for dealing with environmental stresses [107] and mediating nitrogen use efficiency [108]. Lysine is also found to be as a source of energy when carbon is depleted under drought stress in Arabidopsis and tobacco [109]. Two genes encoding lysine catabolism enzymes under osmotic stress in rapeseed (*Brassica napus*) were identified to be coexisting with proline biosynthesis gene which implies that lysine may play a role in withstanding osmotic stress [110]. Therefore, reduced compartmentalization of useful amino acids and activation of selective amino acid transporters for signaling, energy, or stress protection in response to Spd treatment may play a role in Spd-induced drought protection of creeping bentgrass.

Members of the ATP-binding cassette (ABC) family are membrane-bound proteins that participate in transporting a wide range of molecules within several categories (eg. A to H) [111]. A gene encoding one member of the ABC transporters, ABCG protein, was down-regulated in Spd treated plants by 5.1-fold while no regulation was found due to drought. ABCG is known to be involved in transporting ABA to regulate stomata conductance [101], cytokinins to mediate growth [112], and cuticle precursors into the apoplast for cuticular wax deposition [113]. Down-regulation of an ABC transporter detected only in Spd treated plants, demonstrates there may be a regulatory effect of Spd on ABCG transporters. ABC transporters, such as ABCG, are driven by ATP hydrolysis acting as exporters and importers which is energy expensive [114]. Vacuolar membrane-localized Arabidopsis ABCC1 can detoxify folate by importing it into vacuole [114]. Spd treated plants could benefit from less energy expenditure by a reduced number of these transporters; however, it is not clear how PA-induced regulation of ABC transporters may play a role in the drought response of creeping bentgrass.

Transporters are also involved in mediating signaling transducers. One example is that spikes of free calcium ions decoded by calcium binding proteins involves in calcium signaling and leads to a signal amplification or physiological change to adapt to changing environmental conditions [115]. Enrichment (543/341) of calcium binding proteins was identified. Expression of gene encoding calcium-binding mitochondrial carrier protein SCaMC-1 was up-regulated by 5.1-fold when compared to plants treated with Spd under drought (WS vs DS). This transporter functions as an ATP importer in the mitochondria and S-Adenosyl methionine transporter in plastid to cope with stress in Arabidopsis [115]. It also acts as cell traffic mediator in sweet orange seedlings (*Citrus sinensis*) under boron deficiency [116]. Up-regulation of these calcium involved transporters might present another strategy of Spd mediated drought tolerance by triggering calcium induced signaling pathways.

#### Signaling processes

Plants undergo a series of signaling transduction via various interactions among phytohormones and downstream signaling transducers for drought tolerance. A drought tolerant model revealed by the action of PA was proposed by Hatmi et al. [117] in grapevine (Vitis vinifera) in which PA homeostasis was regulated to trigger downstream defense pathways through signal transduction. Pál et al. [118] also indicated that PAs cross-talk with NO, H_2_O_2_, and Ca^2+^, which mediate other phytohormones and signaling molecules to promote abiotic stress defenses. In response to Spd treatment, a DE gene in a calcium-mediated signaling pathway was down-regulated (-6.8 fold) when compared between drought Spd treated and well-watered plants (WS vs DS). Transgenic Arabidopsis lines with increased endogenous Put and Spm exhibited drought tolerance by means of cross-talk with ABA, Ca^2+^, and other hormonal pathways [11]. Over expressing SAMDC1 to increase endogenous Spm content in Arabidopsis induced up-regulation of a gene encoding an ABA biosynthesis gene (NCED), and those plants were more salt tolerant [11] The phenotype of hypersensitivity to salinity stress in Spm deficient Arabidopsis mutant (acl5/spms) is similar to Arabidopsis that over expresses a gene encoding a Ca^2+^/H^+^ antiporter. This Spm mutant performs poorly in Ca^2+^ deficient media, which indicates the close relationship between Spm and Ca^2+^ [119]. Pottosin and Shabala [120] further showed that Ca^2+^ influx across plasma membranes was induced by oxidation of PAs at the apoplast with Ca^2+^ as the second messenger to regulate stomata movement to promote drought tolerance. Here we found one DE gene associated with Ca^2+^ signaling and we found that it was down-regulated due to Spd treatment. As Ca^2+^ is involved in signaling a myriad of different processes in the plant in addition to stomatal regulation, it is unclear whether Spd may affect Ca^2+^ mediated signaling that is associated with drought tolerance in this study.

Cross-talk among the hormones ABA, ethylene, and GA are known to play an important role in stress signaling [121]. Limited information is available regarding how PAs may interact with phytohormone metabolism to affect drought tolerance, in particular GA. Enriched transcripts (3595/2287) associated with GA signaling were detected in response to Spd application in creeping bentgrass. Expression of a gene encoding a GA regulated protein 2 was up-regulated (2.1 fold) when compared between drought and well-watered plants with Spd treatment (WS vs DS). Comparatively, the extent of up-regulation for this gene under drought (WC vs DC) was 7.5 fold. It is clear there is a connection between PAs and GA signaling. Dwarfism caused by increased Put content in transgenic Arabidopsis was rescued by exogenous GA application [122]. Shukla et al. [8] found that Spd treatment may promote creeping bentgrass tillering rates and leaf number compared with the non-Spd treated plants. It is not yet clear how GA signaling may be associated with PA function. Further evaluation of Spd regulation of GA mediated signaling for drought tolerance will enhance our understanding of GA and PAs in drought tolerance.

#### Stress defense

Antioxidants are a major part of the plant defense mechanisms under various environmental stresses since they scavenge ROS produced under stress to reduce damage to cellular constituents [123]. Effective regulation and maintenance of antioxidant systems can play a major role in the drought tolerance of creeping bentgrass and other perennial grass species [3, 5, 6, 44]. In some plant tissues, PAs may act directly as antioxidant agents [124]. Foliar Spd application in creeping bentgrass has been shown to reduce lipid peroxidation and enhance drought tolerance. Li et al. [9] detected an up-regulation of peroxidase in ‘Penn-A4’ creeping bentgrass after exogenous Spd application. At the transcriptional level, we have found enriched transcripts (188/50) that are associated with antioxidants. Expression of a DE gene encoding a cationic peroxidase, SPC4, was up-regulated (2.2 fold) in response to Spd application under drought (WS vs DS). Peroxidase plays an important role in detoxifying H_2_O_2_ under stress and cationic peroxidase SPC4 is one of the peroxidase isoforms in sorghum grain (Sorghum bicolor) [125]. Protein expression of SPC4 in a naturally drought tolerant purple feathergrass (Stipa purpurea) was higher than in the sensitive type, which indicates its importance in drought tolerance [126]. Additional investigation of PA effects on the regulation of creeping bentgrass antioxidant systems is needed.

Enriched transcripts (46/23) were also found for other antioxidants. A gene encoding glutathione synthetase (GS) in Spd treated plants was up-regulated (8.9 fold) while down-regulation of this process was observed in drought (WC vs DC; -2.7 fold) and drought Spd treatment (WS vs DS; -9.9 fold). GS is one of the regulatory enzymes that catalyze formation of glutathione (GSH) [127]. GSH acts as substrate of glutathione S-transferase for antioxidant system to scavenge free radicals under stress or recycles ascorbic acid from its oxidized form to its reduced form by dehydroascorbate reductase [128]. In addition to the antioxidant effect of GSH, it induces production of H_2_O_2_ and Ca^2+^ for stomata closure in Arabidopsis [129]. Thus, Spd may regulate transcripts involved in antioxidant production to fine tune the antioxidant system to improve drought tolerance.

Chemical priming, such as spraying PAs, is thought to involve epigenetic modification for a plant’s stress memory to enable plants to better survive subsequent stresses [130–133]. In this study, enriched transcripts (426/382) associated with chromatin organization (GO: 0006325) were identified. DE gene encoding histone deacetylase HDT3 was up-regulated by 8.7-fold. Although epigenetic modification occurs in histone or DNA through methylation, acetylation, demethylation, or deacetylation, histone deacetylation might be one of the mechanisms that is promoted in response to applied Spd for drought tolerance. A better understanding of possible epigenetic changes for drought stress related to PAs in plants is still needed.

Processes associated with secondary metabolism can include important stress tolerance mechanisms. A gene encoding a phosphoethanolamine N-methyltransferase 1 (PEAMT) for choline biosynthesis process was up-regulated by 8.3-fold due to drought while this process was down-regulated by 3.3-fold in Spd treated plants (WC vs DS). PEAMT catalyzes the reaction by adding methyl groups for choline formation which serves as the precursor for biosynthesis of plasma membrane and glycine betaine. Enhanced choline and glycine betaine caused increased Arabidopsis osmotic stress tolerance [134] and mutation of PEAMT showed early senescence and susceptibility to salinity stress [135]. A gene encoding 1-deoxy-D-xylulose-5-phosphate synthase (DXS) was up-regulated by 2.7-fold while it was down-regulated in Spd treated plants by 3.7- fold. DXS is the committed enzyme catalyzes the first step of isopentenyl diphosphate (IPP) formation within methylerythritol-4-phosphate pathway for terpene biosynthesis. In white grape, IPP derived compounds like phenylpropanoids, monoterpenes, and tocopherols were detected due to drought through transcriptome analysis [136]. Alkaloids, flavonoids [137], and anthocyanins [138–139] in pea (*Pisum sativum*) were induced by drought stress and were thought to act as non-enzymatic antioxidants. Therefore, Spd treated plants may not have been experiencing as much cellular stress damage. Spd treatment may have reduced the costs associated with the production of secondary metabolites that may be needed to reverse cellular stress damage incurred by creeping bentgrass under drought stress.

## Conclusions

A fully sequenced genome for creeping bentgrass is not yet available. RNA-Seq analysis coupled with functional annotation was successfully used to identify differentially expressed genes due to drought stress and Spd treatment in creeping bentgrass. This study provides insight into gene transcripts and predicted functions in creeping bentgrass due to exogenous Spd application in response to drought. PA treatment primarily affected energy metabolism such as by transcripts associated with photosynthetic processes, triggered stress defenses such as antioxidant pathways, and other metabolic pathways under drought conditions. As transcript levels can only suggest possible changes in protein expression, physiology, or biochemistry, future work is needed to more directly associate plant responses with the transcriptome changes found here due to PA and drought treatment. Specifically, PA regulation of phytohormones, carbon fixation processes, carbohydrate allocation and translocation, metabolomics, and proteomic studies may be beneficial to better understand PA effects on plants under stress.

## Supporting information

S1 Fig KEGG map for arginine biosynthesis. (PDF)

S2 Fig KEGG map for nitrogen metabolism. (PDF)

S3 Fig KEGG map for glycolysis. (PDF)

S4 Fig KEGG map for carbon fixation in photosynthesis. (PDF)

S5 Fig KEGG map for starch and sucrose metabolism. (PDF)

S1 Table Reads trimming summary. (XLSX)

S2 Table All transcripts abundance estimation. (XLS)

S3 Table Filtered raw read counts across all samples. (XLSX)

S4 Table Primer pairs for qRT-PCR. (XLSX)

S5 Table Reads alignment efficiency. (XLSX)

S6 Table Over represented GOs. (XLSX)
