# Supplementary material for: Transcriptome analysis of creeping bentgrass exposed to drought stress and polyamine treatment
Source: PLoS One. 2017 Apr 26;12(4):e0175848. doi: 10.1371/journal.pone.0175848 (PMC5406032; doi:10.1371/journal.pone.0175848)
Supplement: S1 Fig — (PDF) [file pone.0175848.s001.pdf]

Transcriptome Analysis of Creeping Bentgrass Exposed to Drought Stress and Polyamine Treatment

Yingmei Ma, Vijaya Shukla, and Emily B. Merewitz

SUPPORTING INFORMATION

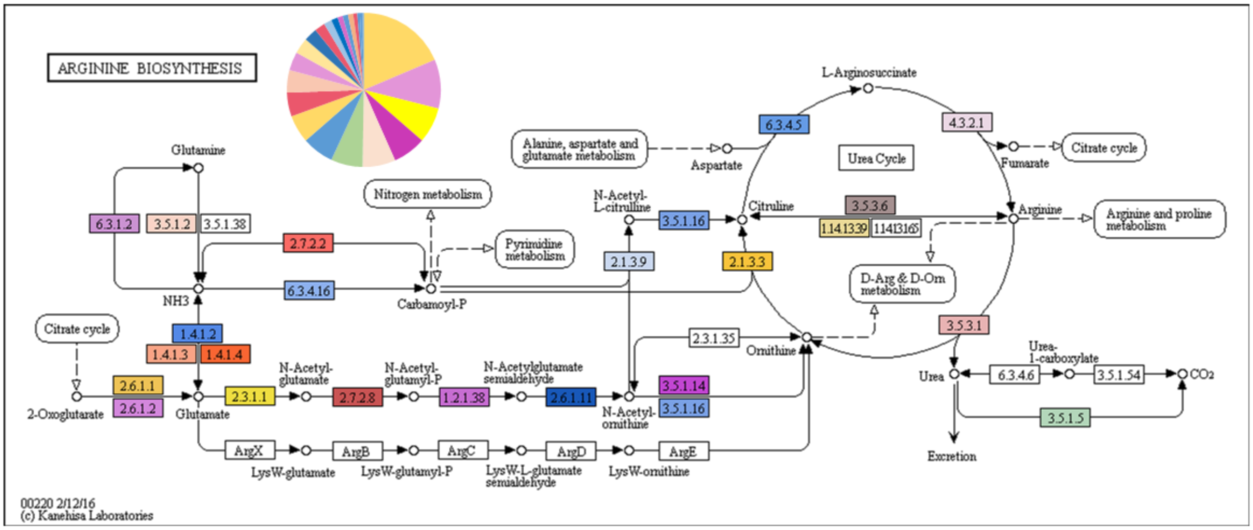

S1 Fig. An example of the KEGG metabolic pathway map showing arginine biosynthesis. The color shaded boxes indicated the enzymes code (EC) number that is encoded by the transcripts detected in creeping bentgrass and in the genome of the KEGG pathway maps by comparing this enzyme coding gene sequence with the reference sequences. Circle is a metabolic compound in the KEGG pathway map. Number of sequences detected for enzymes with color shaded boxes was presented in the pie chart.
